# Transcriptional Programs and Regeneration Enhancers Underlying Heart Regeneration

**DOI:** 10.3390/jcdd6010002

**Published:** 2018-12-22

**Authors:** Ian J. Begeman, Junsu Kang

**Affiliations:** Department of Cell and Regenerative Biology, University of Wisconsin School of Medicine and Public Health, University of Wisconsin–Madison, Madison, WI 53705, USA; begeman@wisc.edu

**Keywords:** heart, zebrafish, regeneration, enhancer, transcription, gene regulation, development

## Abstract

The heart plays the vital role of propelling blood to the entire body, which is essential to life. While maintaining heart function is critical, adult mammalian hearts poorly regenerate damaged cardiac tissue upon injury and form scar tissue instead. Unlike adult mammals, adult zebrafish can regenerate injured hearts with no sign of scarring, making zebrafish an ideal model system with which to study the molecular mechanisms underlying heart regeneration. Investigation of heart regeneration in zebrafish together with mice has revealed multiple cardiac regeneration genes that are induced by injury to facilitate heart regeneration. Altered expression of these regeneration genes in adult mammals is one of the main causes of heart regeneration failure. Previous studies have focused on the roles of these regeneration genes, yet the regulatory mechanisms by which the expression of cardiac regeneration genes is precisely controlled are largely unknown. In this review, we will discuss the importance of differential gene expression for heart regeneration, the recent discovery of cardiac injury or regeneration enhancers, and their impact on heart regeneration.

## 1. Introduction

The ability to regenerate damaged cardiac tissues varies across vertebrate species. While neonatal mice can regenerate injured hearts during the first week of life, adult mammalian hearts regenerate poorly and form scar tissue instead. Scar formation impedes the ability of the heart to propel blood to the whole body, leading to increased morbidity and mortality [1]. Unlike adult mammals, adult nonmammalian species including zebrafish, goldfish (*Carassius auratus*), giant danio (*Devario aequipinnatus*), newts, axolotls, and frogs can regenerate injured hearts [2,3,4,5,6,7]. Thus, investigating these models to understand heart regeneration can provide insights into heart repair that may be applied to mammals.

Heart regeneration studies using zebrafish and mice have revealed multiple factors influencing cardiac regeneration. Zebrafish live in an aquatic environment that has a low oxygen level, and this hypoxic condition likely enables the proliferation of cardiac muscle cells, or cardiomyocytes (CMs) [8]. The number of complete sets of chromosomes also affects CM proliferation. While nonregenerative hearts, such as adult mammalian hearts, are composed of CMs that are primarily polyploid (containing more than two sets of homologous chromosomes), almost all zebrafish CMs are diploid (containing two sets of homologous chromosomes) [9]. Notably, the percentage of diploid CMs is highly variable across mouse strains and correlates with the ability to enhance post-injury CM proliferation in adult mice [9]. Interestingly, genetically modified zebrafish in which a majority of CMs are polyploid fail to regenerate, indicating that CM polyploidization acts as a heart regeneration barrier [9,10]. In addition to environmental and cellular structure discrepancies, other main determinants of cardiac regenerative capacity are differentially expressed regeneration-driving genes [11,12,13,14]. Transcriptomic analysis of CMs, fibroblasts, leukocytes, and endothelial cells from infarcted and non-infarcted neonatal (P1) and adult (P56) mice has revealed that adult, but not neonatal, CMs and endothelial cells fail to reactivate proliferation-related genes upon cardiac injury, which contributes to the loss of cardiac regeneration in the adult stage [13]. This study demonstrated that regulatory programs governing neonatal cardiac regeneration may be altered in adults to prevent the reactivation of regeneration gene expression. Thus, transcriptional regulation and underlying regulatory mechanisms are crucial for heart regeneration. In this review, we first describe how the transcription of regeneration genes implicates heart regeneration in multiple species. We then highlight recent discoveries of regulatory elements controlling the transcription of regeneration genes: cardiac regeneration enhancers.

## 2. Differentially Expressed Genes Underlie Maintenance or Loss of Cardiac Regenerative Capacity

**Figure 1:**
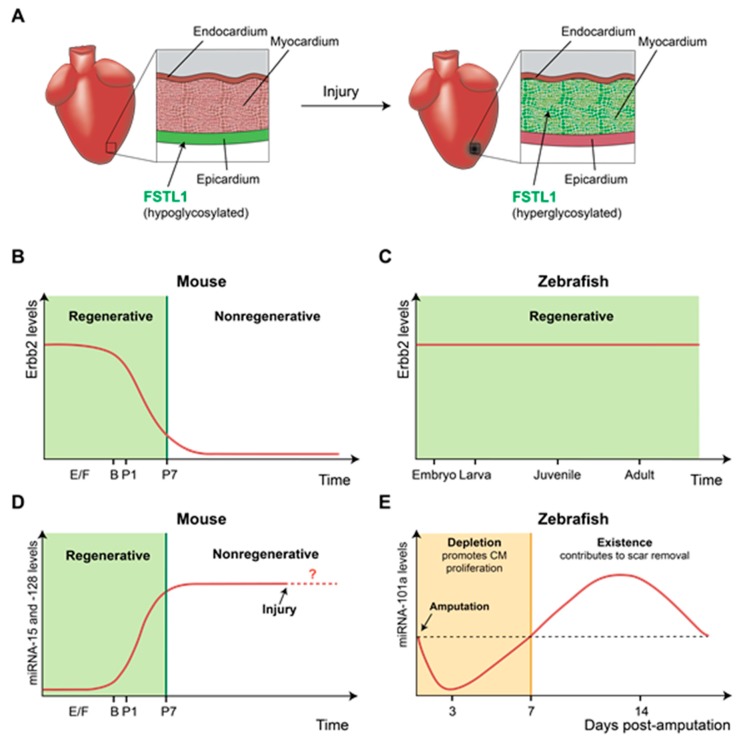
Differential gene expression affects regenerative ability. (**A**) *Fstl1* expression in the mouse heart is altered upon injury. In uninjured hearts, the epicardium produces *Fstl1*, which has the potential to promote cardiomyocyte (CM) proliferation. However, in injured hearts, *Fstl1* is mainly produced by the myocardium rather than the epicardium. *Fstl1* derived from the myocardium appears to be unable to promote CM proliferation due to glycosylation. (**B**) The level of *Erbb2*, a receptor of *Nrg1*, declines after birth, resulting in poor regenerative capacity in the mouse heart. (**C**) In zebrafish, *Erbb2* expression is maintained throughout life, and a sustained *Erbb2* level contributes to retaining cardiac regenerative capacity in the adult stage. (**D**) The expression level of the miRNA-15 family and miRNA-128 increases after birth in mice to inhibit heart regeneration. However, it is unclear whether expression of these miRNA is maintained upon injury. (**E**) The expression level of miRNA-101a is dynamic during heart regeneration in adult zebrafish. Depletion of miRNA-101a in the early regenerative stage promotes CM proliferation, while the presence of miRNA-101a in the late regenerative stage contributes to scar tissue removal. The ability to precisely modulate miRNA expression during heart regeneration enables zebrafish to retain remarkable regenerative capacity. In (**B**,**D**): E/F, embryonic/fetal period; B, birth; P, postnatal period.

*Follistatin-like 1* (*Fstl1*) was discovered as a potent CM mitogen secreted from an epicardial mesothelial cell line [15]. Delivering *Fstl1* protein via an epicardial patch stimulates cardiac regeneration in injured mouse and swine hearts, thereby improving cardiac function. In uninjured mouse hearts, *Fstl1* is typically produced by the epicardium, the outermost layer covering the cardiac chambers. However, cardiac injury in mice downregulates *Fstl1* expression in the epicardium but strongly induces *Fstl1* expression in the myocardium. Interestingly, unlike epicardium-derived *Fstl1*, CM-derived *Fstl1* does not have the potential to promote CM proliferation, possibly due to hyperglycosylation [15]. As shown by this study, cellular sources of regeneration factors that are altered upon injury can affect post-translational modification, resulting in different mitogenic effects from regeneration factors (Figure 1A). An interesting question is how and what regulatory mechanisms control the expression of the same target gene in distinct cell types upon injury.

*Neuregulin 1* (*Nrg1*) is another potent CM mitogen in mammals, as demonstrated in neonatal mice and cultured human myocardium, and NRG1 administration has been proposed as a therapeutic strategy to promote cardiac regeneration [16,17,18,19]. The cardiomyogenic effect of NRG1 was tested with neonatal mouse hearts and pediatric patient-derived myocardium [16]. Interestingly, NRG1 therapy is most effective at an early age, such as 3 days in neonatal mice and 6 months in diseased human heart cells; however, the therapeutic effect significantly declines thereafter [16]. Another independent study demonstrated that *Nrg1* effectively induced CM proliferation before postnatal day 7 (P7) in mice, but its effects diminished thereafter [18]. The limited time window of *Nrg1*-induced regeneration is determined by the expression level of *Erbb2*, a *Nrg1* receptor, in CMs, as *Erbb2* expression is sharply downregulated in neonatal mice after P7 (Figure 1B). In injured adult mouse hearts, transient induction of active *Erbb2* can restore the ability to regenerate CMs, supporting the positive influence of *Erbb2* levels on NRG1/ERBB2 signaling-mediated cardiac regeneration.

*Nrg1* also has a mitogenic effect in adult zebrafish hearts [20]. Upon cardiac injury, *Nrg1* expression in adult zebrafish is upregulated in multiple cells, including regulatory T cells [21] and perivascular cells of ventricular wall [20], to stimulate CM proliferation. In addition, overexpression of *Nrg1* in uninjured hearts triggers CM proliferation, causing cardiomegaly through persistent addition to the cardiac muscle wall [20]. Unlike adult mammals, adult zebrafish maintain *Erbb2* expression in the heart, which contributes to retaining cardiac regenerative capacity in injured hearts and stimulating CM proliferation in response to ectopic *Nrg1* expression in uninjured hearts (Figure 1C). Together with mouse data, these results illustrate that losing the capability to express regeneration genes in aged mammalian hearts may act as a roadblock to heart regeneration. Further experiments to define the regulatory mechanisms suppressing *Erbb2* expression in mouse hearts after P7 or those maintaining *Erbb2* levels in adult zebrafish hearts will lead to the identification of new molecular targets for heart repair.

MicroRNAs (miRNAs), a class of small noncoding RNAs that play roles in RNA silencing and posttranscriptional regulation [22], are one of the main regulators of cardiac regeneration. In mice, the miRNA-15 family and miR-128 are expressed at low levels shortly after birth, but their expression is robustly increased at P7, a time point at which CMs lose their regenerative capacity [23,24]. The expression pattern of these miRNAs as well as their negative role in heart regeneration was revealed by functional studies, showing that overexpression of these miRNAs in neonatal mice inhibits CM proliferation and that their depletion at the adult stage enhances CM proliferation. Neonatal mouse hearts appear to downregulate the expression of several miR-15 family members in response to cardiac injury, revealing the presence of regulatory mechanisms that may hamper the negative effects of miRNA on heart regeneration (Figure 1D) [23].

Adult zebrafish hearts also express inhibitory miRNAs, such as miR-101a and miR-133, in uninjured hearts [25,26]. Cardiac miR-101a expression is profoundly reduced in response to cardiac injury and steadily declines until 3 days post-amputation (dpa), when CMs dedifferentiate and start to proliferate. Subsequently, miR-101a expression significantly increases above the level of uninjured hearts by 7–14 dpa before returning to uninjured levels at 30 dpa. The dynamic miR-101a level during heart regeneration appears to be essential, as shown by functional analysis; miR-101a depletion at the onset of cardiac injury enhances CM proliferation whereas the sustained reduction in miR-101a after injury prevents scar tissue removal [25]. These studies suggest that adult zebrafish hearts maintain the regulatory mechanisms, by which miR-101a expression is precisely modulated to facilitate heart regeneration (Figure 1E). Due to a lack of data describing the endogenous levels of miRNA-15 and miRNA-128 in adult mouse hearts upon injury, it is unclear whether the absence of the ability to downregulate inhibitory miRNA expression upon injury contributes to nonregenerative hearts. However, possessing a regulatory program controlling dynamic miRNA expression is undoubtedly one factor for maintaining cardiac regenerative capacity.

Immune cells also play important roles during heart regeneration. While inflammation triggers nonregenerative scarring in adult mice after cardiac injury, acute inflammation is required for cardiac regeneration and drives CM proliferation in neonatal mice [27]. These different inflammation effects on heart regeneration may be caused by the differing compositions of macrophage subsets in regenerating adult and neonatal hearts [28,29]. The majority of neonatal cardiac macrophages originate from the embryonic yolk sac and reside in the heart. Upon cardiac injury, these resident heart macrophages mediate angiogenesis and regeneration without scar formation. By contrast, in adult hearts following cardiac injury, the resident macrophages are not proliferative but are instead replaced by infiltrating proinflammatory monocytes and monocyte-derived macrophages, resulting in scar formation. Several studies have demonstrated that macrophages and monocytes produce regenerative factors, including interleukin-6 (IL-6) and myeloid-derived growth factor (MYDGF), which contribute to the regenerative capacity of hearts [30,31]. These studies suggest that distinct gene expression profiles in immune cells also affect heart repair.

Collectively, these studies have provided evidence that preserving regulatory functions to ensure regeneration factor expression constitutes the core mechanism of heart regeneration. Thus, the next critical question is where the regulatory DNA elements are in the genome and which molecular components modulate their activity.

## 3. Enhancers Are Key Regulatory Elements Controlling Cardiac Gene Expression and Function

Enhancers are key *cis*-regulatory DNA elements controlling spatiotemporal gene expression [32,33]. The number of enhancers is expected to surpass the number of protein-coding genes, as up to one million putative enhancers have been identified in the human genome [34,35], suggesting that multiple enhancers regulate the expression of a single gene in various circumstances. Enhancers often contain clusters of sequence-specific transcription factor binding motifs, and cooperative interactions of binding factors at the enhancer locus drive target gene expression in a specific context. Active enhancers are characterized by accessible DNA regions, which are devoid of conventional nucleosomes, and the presence of specific histone modifications, including histone H3 lysine 4 monomethylation (H3K4me1) and H3K27 acetylation (H3K27ac) [34]. The most common methods to predict active enhancer candidates include mapping accessible chromatin landscape using DNase I hypersensitive site sequencing (DNase-seq) [34] or assaying for transposase-accessible chromatin using sequencing (ATAC-seq) [36], and analyzing genome-wide profiles of active enhancer markers, such as H3K24me1 and H3K27ac using chromatin immunoprecipitation followed by deep sequencing (ChIP-seq) [37,38]. The in vivo functionality of enhancers can be validated by a transgenic assay, in which enhancer sequences coupled with a minimal promoter and a reporter gene are introduced into an animal to test reporter gene expression in a context of interest [39]. In recent years, several groups have also validated enhancer activity by enhancer deletions using genome editing followed by examining the disruption of target gene expression and the phenotype [40,41,42].

Multiple studies have been performed to map cardiac enhancer landscapes in developing and adult mammalian hearts. A multitiered computational analysis, using experimentally validated cardiac enhancers identified a cluster of sequence features shared by cardiac enhancers, with which more putative cardiac enhancers throughout the genome were selected. Further in vivo transgenic enhancer assays using zebrafish and mice verified that significant numbers of putative enhancers displayed activity in hearts [43]. Using ChIP-seq with p300/CREB-binding protein (CBP), transcriptional coactivators associated with enhancers, Blow et al. identified over 3000 cardiac enhancer candidates from embryonic mouse cardiac tissue, and a significant portion of the selected candidates exhibited in vivo cardiac activity [44]. An experimentally defined genome-wide map of human heart enhancers was also generated by ChIP-seq experiments of p300/CBP [45]. Distally localized p300/CBP peaks were markedly enriched near genes associated with cardiovascular development, function, and diseases, suggesting that these sequences may control the expression of cardiovascular genes. Transgenic mouse reporter assays with 65 enhancer candidates revealed that the majority of these cardiac enhancer candidates were authentic in vivo heart enhancers [45], indicating their potential roles in cardiac development and diseases.

Recent work has provided evidence of the importance of cardiac enhancers for cardiac function. Dickel et al. generated a comprehensive genome-wide catalog of cardiac enhancers in mammals by integrating nearly all the available epigenomic profiles of hearts, ranging from prenatal to adult stages of human and mouse heart samples [40]. This cardiac enhancer meta-analysis defined over 80,000 putative cardiac enhancers, and a subsequent retrospective approach matched these cardiac enhancers with the developmental stages in which they are potentially active. In addition to the transgenic enhancer assay, the investigators employed genome editing to delete cardiac enhancer sequences and then assessed the necessity of heart enhancers for cardiac development and function. Two different cardiac enhancers, mm77 and mm771, upstream of *Myl2* and *Myh7*, respectively, were selected because of their association with cardiac diseases in humans. Interestingly, loss of either mm77 or mm771 resulted in decreased expression of *Myl2* or *Myh7*, respectively, and led to abnormal cardiac organization and reduced cardiac function [40]. These results highlight the importance of cardiac enhancers for cardiac health.

Cardiac enhancer studies have shown that enhancers are key regulators that maintain cardiac function. Thus, questions have arisen regarding whether there are regeneration-associated enhancers responsible for cardiac injury and how their activity is regulated.

## 4. Epicardial Enhancers Drive Epicardial Factor Expression in Developing and Injured Hearts

The epicardium plays crucial roles during cardiac development by providing multipotent progenitor cells for cardiac fibroblasts, vascular smooth muscle cells, adipocytes, pericytes, endothelium, and endocardium, while some results are controversial [46,47,48,49,50]. The epicardium is also a vital tissue that promotes cardiac repair. Following cardiac injuries, epicardial cells contribute to the generation of various cardiac cells, including vascular smooth muscle cells, perivascular cells, and myofibroblasts [49,51,52,53]. In addition, cardiac injuries rapidly activate the epicardium in an organ-wide manner to re-express embryonic developmental genes such as *retinaldehyde dehydrogenase 2* (*Raldh2*), *T-box transcription factor 18* (*Tbx18*), and *Wilms tumor 1* (*Wt1*), and the activated epicardium acts as a paracrine center to stimulate CM proliferation [54,55,56]. Although genetic factors activated in the epicardium are well described, the upstream regulation of epicardial gene activation during development or in response to cardiac injury has just begun to be understood.

**Figure 2:**
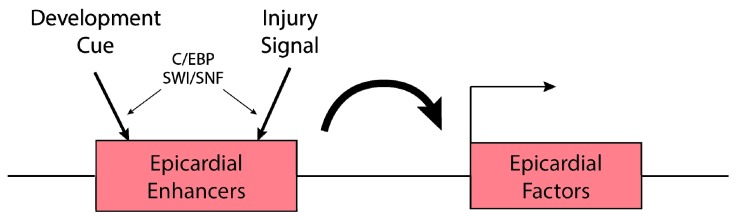
Epicardial enhancers are activated by development cues and cardiac injury. Several evolutionarily conserved regions near epicardial factors are activated during development and upon cardiac injury to drive gene expression in epicardium. C/EBP and the SWI/SNF complex mediate epicardial enhancer activation.

Multiple epicardial genes, such as the *Tbx18*, *transcription factor 21* (*Tcf21*), *Raldh2*, and *Wt1*, are highly enriched in both embryonic and injured adult epicardium [57]. To identify the regulatory elements governing their epicardial expression, Huang et al. surveyed enhancer activity of 39 evolutionarily conserved regions (CRs) associated with the epicardial genes. Through screening with cultured and in vivo mouse hearts, the investigators demonstrated that *Raldh2* CR2 and *Wt1* CR14 were capable of directing epicardial gene expression [58]. Further analysis demonstrated that CCAAT/enhancer binding protein (C/EBP) transcription factors bound to *Raldh2* CR2 and *Wt1* CR14 epicardial enhancers, and together with HOX, MEIS, and Grainyhead transcription factors, established a transcriptional network for embryonic gene expression in the epicardium. Notably, *Raldh2* CR2 and *Wt1* CR14 enhancers are also activated in adult mouse hearts in response to myocardial infarction (MI) [58], suggesting that embryonic epicardial enhancers are reactivated to repurpose the developmental gene program in adult hearts after injury (Figure 2).

Another independent study revealed the epigenetic regulation of epicardial activation. *Brahma-related gene 1* (*Brg1*) and *Brahma* (*Brm*), catalytic subunits of the Switch/Sucrose nonfermentable (SWI/SNF) chromatin-remodeling complex, are expressed in the epicardium of developing and injured adult mouse hearts [59]. BRG1 binds to several evolutionarily conserved regions (ECRs) in the *Wt1* locus that have been identified as epicardial enhancers of both developing and injured adult mouse hearts. BRG1 recruitment to *Wt1* epicardial enhancers is mediated by C/EBP-beta, and *Brg1* and *C/EBP-beta* are required for *Wt1* activation in the epicardium of developing and injured hearts (Figure 2) [58,59]. This work demonstrates that injury cues activate the ATP-dependent SWI/SNF chromatin-remodeling complex, which regulates chromatin accessibility to induce transcription of injury-responsive genes. Collectively, epicardial enhancer studies in mice have provided evidence of the presence of epicardial enhancers in both developing and injured hearts, implying that developmental enhancers can be reactivated to drive injury-induced expression.

## 5. Cardiac Tissue Regeneration Enhancers in Zebrafish Ensure Regeneration-Specific Expression

An intriguing question is whether cardiac injury activates regeneration-specific enhancers to establish and/or maintain genetic programs supporting regeneration. To address this question, the zebrafish model offers exceptional experimental advantages. Unlike adult mammals, adult zebrafish possess a remarkable capacity to regenerate injured hearts, making zebrafish an ideal model for studying the regulatory mechanisms of adult heart regeneration [2]. Because transgenesis is relatively simple and easy in zebrafish, a growing number of studies have employed the zebrafish system to test whether nonmammalian and mammalian enhancer candidates exhibit activity in various tissues or organs, including hearts [43,60,61]. In addition, various transgenic lines and genetic drivers are available for directing transgene expression in distinct cardiac cell types, such as CMs and epicardium, allowing the identification of cell type-specific enhancers [49,62].

**Figure 3:**
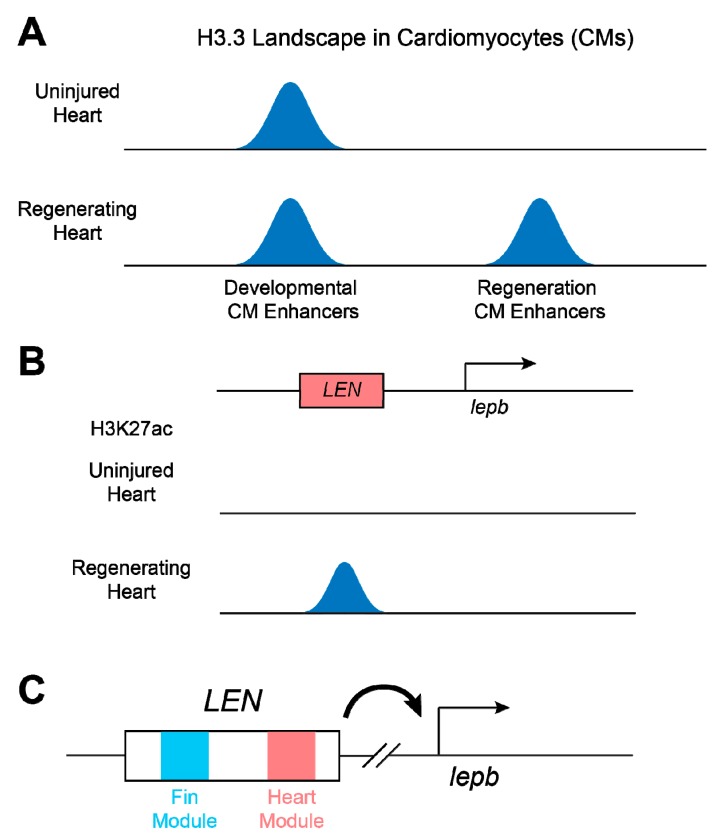
Cardiac regeneration enhancer elements. (**A**) Cardiomyocyte (CM)-specific enhancers in zebrafish. CM-specific histone H3.3 profiling of uninjured and regenerating zebrafish hearts captures regulatory elements preferential for heart development and regeneration. (**B**) ChIP-seq analysis with active enhancer markers, such as H3K27ac, identifies *lepb*-linked regeneration enhancer (*LEN*), which can direct regeneration-specific expression in hearts and fins of zebrafish. (**C**) *LEN* consists of tissue-specific regeneration modules, each of which mediates gene transcription in their target tissues.

Active enhancers are thought to be accessible for sequence-specific transcription factors to bind enhancer regions. Gaining accessibility results in high turnover of the nucleosome by replacing canonical nucleosomes with unstable nucleosomes containing histone variants H2A.Z and H3.3 [63,64,65]. Because the H3.3 histone variant is selectively enriched at transcriptionally active genes, promoters, insulators, and enhancers, a new way has emerged to identify *cis*-regulatory elements, including active enhancers, by analyzing H3.3 profiles [62,66]. In a recent study, Goldman et al. utilized the specificity of H3.3 in the zebrafish system to find CM-specific regeneration enhancers. The investigators created transgenic zebrafish in which a biotinylated H3.3 (H3.3-bio) was specifically expressed in CMs to generate CM-specific H3.3 profiles of uninjured and regenerating hearts [62]. Computational analysis of CM-specific H3.3 profiles identified a massive number of de novo H3.3 peaks associated with regeneration. Transgenic assays with zebrafish carrying the *cis*-regulatory elements marked by H3.3 in regenerating CMs revealed that most of these regeneration-associated H3.3 peaks are cardiac regeneration enhancers directing regeneration-dependent gene expression in myocardium (Figure 3A). Further motif analysis with validated regeneration enhancers predicted potential regeneration modules that may construct core regulatory elements activated in CMs of regenerating hearts [62]. Because the H3.3-bio transgenic system does not require a cell isolation procedure, this approach provides a non-invasive method to obtain cell type-specific enhancer profiles and to reveal cell type-specific regulatory mechanisms in regenerating hearts.

Cardiac regeneration-specific enhancers were also discovered by employing the zebrafish system [67]. Unbiased transcriptomic analysis with regenerating fin and cardiac tissues defined the *leptin b* (*lepb*) gene, one of two zebrafish paralogs related to mammalian *Leptin* [68], as a robustly injury-induced gene in hearts and fins. Interestingly, a *lepb:EGFP* bacterial artificial chromosome (BAC) reporter line containing a 105-kb DNA sequence upstream of the *lepb* start codon barely drove EGFP expression in hearts or fins throughout life, but strongly induced EGFP expression in hearts or fins during regeneration. Surveying active enhancer candidates in *lepb* BAC regions identified two distal fragments that were marked by H3K27ac in regenerating, but not uninjured, hearts. Particularly, a 1.3-kb small fragment among these putative enhancers was capable of directing regeneration-dependent expression without developmental induction, indicating that this *lepb*-linked element is a regeneration-specific enhancer (Figure 3B). Notably, transgenic lines carrying a series of *lepb* regeneration enhancer (*LEN*) sequences revealed that *LEN* consisted of fin- and cardiac-specific regeneration modules [67], providing evidence that different tissue injuries use distinct gene regulatory networks to drive the expression of the same target gene (Figure 3C).

Enhancers rapidly change during evolution, contributing to physiological and morphological diversity [33,69]. Cardiac regeneration enhancers might also evolve rapidly, suggesting that changes in cardiac regeneration enhancers play important roles in diverse cardiac regeneration abilities across species. Zebrafish *LEN* is unlikely to be conserved in mammalian genomes at the sequence level, implying that mammals may have lost regeneration enhancers during evolution, and thus have limited regenerative capacity. However, zebrafish *LEN* can surprisingly drive injury-dependent expression in damaged mouse hearts [67], implying that mammalian gene regulatory networks have the potential to activate zebrafish elements. This result also posits that evolutionary loss of cardiac regeneration enhancers might affect regeneration-driving gene expression and consequently lead to deficient heart regeneration in mammals. This logic can be applied to develop therapeutic strategies to enhance regenerative potential; engineering active cardiac regeneration enhancers in regeneration-deficient animals can awaken dormant expression of regenerative factors and subsequent enhancement of heart regeneration. In zebrafish, coupling *LEN* with *Nrg1* enables regeneration-restricted induction of *Nrg1* in the wound area, and the temporal ectopic *Nrg1* induction enhances CM proliferation [67]. These data provide a proof of concept that regeneration enhancer engineering presents a promising strategy to precisely deliver the CM mitogen to injured hearts. It will be interesting to examine whether delivery of regenerative factors using cardiac regeneration enhancer engineering will overcome the barriers of regeneration-deficient hearts and augment heart repair.

## 6. Conclusions

The differential composition of cardiac regeneration enhancers in the genome can lead to distinct gene expression upon cardiac injury, resulting in diverse levels of heart regeneration ability in vertebrates. Recent advances in genomics have enabled us to identify genome-wide landscapes of active enhancers, demonstrating that ambitious efforts will allow the mapping of all regeneration enhancers across species. Such profiles will provide tremendous benefits for potential applications of regenerative medicine. Comparative genomic analysis combined with cross-species transgenic assays will provide insight into how alterations of regeneration enhancers can lead to a decline (or an increase) in regenerative capacity. Biochemical and computational assays will uncover upstream factors and essential motifs, revealing novel gene regulatory networks underlying heart regeneration. More importantly, when applied to gene therapy, regeneration enhancers derived from animals that can naturally regenerate their hearts can be used to drive regeneration factor expression in injured mammalian hearts. This may allow us to unlock the latent healing power of hearts and ultimately to apply these insights to improving heart repair in humans.
